# A Gene-Set Enrichment and Protein–Protein Interaction Network-Based GWAS with Regulatory SNPs Identifies Candidate Genes and Pathways Associated with Carcass Traits in Hanwoo Cattle

**DOI:** 10.3390/genes11030316

**Published:** 2020-03-16

**Authors:** Krishnamoorthy Srikanth, Seung-Hwan Lee, Ki-Yong Chung, Jong-Eun Park, Gul-Won Jang, Mi-Rim Park, Na Yeon Kim, Tae-Hun Kim, Han-Ha Chai, Won Cheoul Park, Dajeong Lim

**Affiliations:** 1 Animal Genomics and Bioinformatics Division, National Institute of Animal Science, RDA, Wanju 55365, Koreajepakr0105@korea.kr (J.-E.P.); kwchang@korea.kr (G.-W.J.); cocci@korea.kr (M.-R.P.); ks901223@naver.com (N.Y.K.); thkim63@korea.kr (T.-H.K.); hanha@korea.kr (H.-H.C.); wcpark@korea.kr (W.C.P.); 2 Division of Animal and Dairy Science, Chungnam National University, Daejeon 34134, Korea; slee46@cnu.ac.kr; 3 Department of Beef Science, Korea National College of Agriculture and Fisheries, Jeonju 54874, Korea; cky95@korea.kr

**Keywords:** Hanwoo, GWAS, non-synonymous SNP, gene-set enrichment, pathway analysis, marbling, carcass weight, eye muscle area, backfat thickness, imputation

## Abstract

Non-synonymous SNPs and protein coding SNPs within the promoter region of genes (regulatory SNPs) might have a significant effect on carcass traits. Imputed sequence level data of 10,215 Hanwoo bulls, annotated and filtered to include only regulatory SNPs (450,062 SNPs), were used in a genome-wide association study (GWAS) to identify loci associated with backfat thickness (BFT), carcass weight (CWT), eye muscle area (EMA), and marbling score (MS). A total of 15, 176, and 1 SNPs were found to be significantly associated (*p* < 1.11 × 10^−7^) with BFT, CWT, and EMA, respectively. The significant loci were BTA4 (CWT), BTA6 (CWT), BTA14 (CWT and EMA), and BTA19 (BFT). BayesR estimated that 1.1%~1.9% of the SNPs contributed to more than 0.01% of the phenotypic variance. So, the GWAS was complemented by a gene-set enrichment (GSEA) and protein–protein interaction network (PPIN) analysis in identifying the pathways affecting carcass traits. At *p* < 0.005 (~2,261 SNPs), 25 GO and 18 KEGG categories, including calcium signaling, cell proliferation, and folate biosynthesis, were found to be enriched through GSEA. The PPIN analysis showed enrichment for 81 candidate genes involved in various pathways, including the PI3K-AKT, calcium, and FoxO signaling pathways. Our finding provides insight into the effects of regulatory SNPs on carcass traits.

## 1. Introduction

Hanwoo (*Bos taurus coreanae*) is the indigenous premium beef cattle of South Korea. It has been intensively selected for higher meat and carcass quality for the last four decades [1]. It is well known for its extensive marbling, texture, flavor, and juiciness, making it the most economically important species in Korea [2,3]. In spite of its premium price, due to its superior meat quality, Hanwoo beef is very popular amongst both Korean and foreign consumers. The breeding value and the genetic worth of Hanwoo is estimated based on the marbling score (MS), carcass weight (CWT), backfat thickness (BFT), and eye muscle area (EMA) [4]. Though substantial improvement in carcass and meat quality have been achieved, due to market requirement for higher quality, and for improving the economic value of Hanwoo, continuous improvement of economically important trait is required [5,6]. A genome-wide association study (GWAS) is an affordable and powerful tool to discover candidate genes and loci associated with quantitative traits [7]. GWASs in livestock, including in Hanwoo [8,9,10,11], have resulted in remarkable insights into the genetic architecture of carcass traits. Genetic variation in complex traits such as carcass and meat quality traits are, however, due to the contribution of many mutations with small effects [1,12] (polygenic effect). Though some of these mutations have been successfully identified through GWASs, the high significance thresholds required to correct for the multiple testing problem results in the identification of only SNPs with a large effect size [7,12]. Further, a GWAS does not make use of the fact that genes work together in a network, and multi-allelic QTL might not be captured due to the bi-allelic nature of SNPs [13]. Moreover, epistasis is an important genetic component underlying phenotypic variation that also accounts for missing heritability [14]. Therefore, a GWAS alone might result in only limited understanding of the nature of complex traits [13]. Suggested solutions to overcome this limitation and understand the genetic complexities regulating complex traits are to complement a GWAS with gene-set enrichment, a protein–protein interaction network (PPIN), and pathway analyses [15,16,17,18]. In GSEA and pathway analysis, a group of related genes harboring significant SNPs identified through a GWAS is tested for enrichment in a specific pathway. While PPIN, which is defined as “Functional epistasis”, looks for an interaction between proteins (genetic elements) within the pathway or with proteins that form complexes with one another [19,20]. Dadousis et al. [13,21] had used the results of a GWAS to provide biological insights into the pathways affecting milk composition and cheese-producing ability in dairy cattle through GSEA and pathway analysis. In those studies, they had used a nominal post GWAS threshold of *p* < 0.05 for generating a list of the top associated SNPs that were then annotated to genes, and which were then used for identifying pathways significantly enriched in the targeted traits. Therefore, supplementing a GWAS with the abovementioned analyses could lead to a better understanding of the mechanisms regulating complex traits [22].

While GWAS identifies many traits associated SNPs, since most of them are in non-coding region, it is hard to map them to biological processes that are crucial for understanding complex traits [23]. In fact, the most important polymorphic loci are those that cause protein-coding differences and can regulate gene expression [24]. Moreover, non-synonymous SNPs change the genetic and amino acid sequence, which might have a deleterious effect on protein function; such mutations are likely to have a phenotypic effect [12]. Most of the SNPs that affect gene expression are reported to fall very close to the gene [25,26]. Hindorff et al. [27] and Kindt et al. [28] had reported that non-synonymous (missense) and promoter SNPs (5kb-promoter region) were significantly overrepresented in association studies, suggesting that regulatory proteins coding SNPs (non-synonymous SNPs + promoter SNPs) might be enriched for trait-associated variants. Therefore, the objective of this study was to use sequence-level imputed SNP data to perform a GWAS with regulatory SNPs (protein coding promoter and non-synonymous (missense) SNPs) and complement it with GSEA, PPIN, and pathway analysis to identify the candidate genes and pathways associated with CWT, BFT, MS, and EMA in Hanwoo cattle. We also estimated the marker effects, variance components, and heritability captured by the regulatory SNPs for the four complex traits. 

## 2. Materials and Methods 

### 2.1. Animals and Phenotypes

A total of 10,215 Hanwoo steers born between 2006 and 2016 on farms across South Korea were used in this study. All the steers were the progeny of 324 sires and 8331 dams (1–3 progenies per dam). Relevant guidelines formulated by the Institutional Animal Care and Use Committee (IACUC) of the National Institute of Animal Science (NIAS), Korea, were followed. The animals were fed a mixture of concentrate and rice straw and were slaughtered between 17 and 62 months of age. The carcass and meat quality traits analyzed in this study were carcass weight (CWT), backfat thickness (BFT), eye muscle area (EMA), and marbling score (MS). Traits were measured as described by Bhuiyan et al. [29]. A descriptive statistical summary of the phenotypes are given in Table 1.

### 2.2. SNP Genotyping, Imputation, and Filtering for Regulatory SNPs

Genomic DNA was isolated from 10,215 Hanwoo tissue samples using the DNeasy Blood and Tissue Kit (Qiagen, Valencia, CA, USA). After checking for quality and quantity on a Nanodrop 1000 (Thermo Fisher Scientific, Wilmington, DE, USA), the samples were genotyped on a Customized Hanwoo SNP50 BeadChip (58,990 SNPs). Only autosomes were included in the analysis. The genotypes were then phased using Eagle v.2.3.2 [30] and then imputed, one chromosome at a time, using Minimac3 [31], to a high-density level, using a reference population consisting of 480 animals genotyped with Bovine HD BeadChip (777,962 SNPs). They were then imputed to the sequence level one chromosome at a time, using whole genome sequence data of a reference population of 311 progeny tested Hanwoo bulls [1], resulting in a total of 27,980,473 SNPs; the imputation pipeline followed for imputing from 770K to the sequence level was the same as the one used for 50K to HD imputation. Following a previous study [32], only SNPs with Minimac3 imputation quality statistic (R^2^) higher than 0.4 were retained for further analysis, resulting in a total of 12,980,473 SNPs. The overall average imputation accuracy, post quality control (R^2^) was 0.76, which was similar to a previously reported study [33].

The physical position of the imputed SNPs was determined using the bovine genome assembly UMD3.1 [34] and the SNPs were annotated with SnpEff version 4.3 [35]. The SNPs were then filtered with SnpSift [36] to include only non-synonymous (missense) SNPs and protein coding SNPs within 5-kb upstream of a gene. These SNPs were considered as regulatory SNPs due to their effect on the protein structure and gene expression [26,37]. The SNPs were further filtered for quality with PLINK v.1.9 software [38] under the following criteria: minor allele frequency (MAF) < 0.01, genotype call rate < 0.10, individual call rate < 0.10, and Hardy–Weinberg equilibrium <0.000001. This resulted in a final set of 450,062 SNPs and 9693 animals for further analysis. All relevant data generated in this study are available within the paper or in the supplementary files. The SNP file generated in this study is freely available for download from National Agricultural Biotechnology information center (www.nabic.rda.go.kr) under the accession no NV-0622.

### 2.3. Genome-Wide Association Analysis, Heritability, and Variance Component Estimation

A genome-wide association study (GWAS) was performed using a mixed linear model implemented in GCTA v.1.91.4 beta3 [39]. Farm (1419), birth year (2006–2016), birth month (1–12), slaughter year (2008–2018), slaughter month (1–12), age (17–62 months), slaughter place (53), sire (324), and dam (8331) were tested for fixed effects. Fixed effects that were significant for all the traits were fitted in the model; this included farm, birth year, slaughter year, slaughter place, age, and sire. The single trait model used was as follows:

y=a+bx+g+e

where *y* is the phenotype; *a* is the mean; *b* is the additive effect (fixed effect) of the candidate SNP to be tested for association; *x* is the SNP genotype coded as 0, 1, or 2; *g* is the accumulated effect of all the SNPs captured by the GRM (genetic relationship matrix, calculated using all the SNPs), and *e* is the residual effect. Bonferroni corrections were applied to correct for multiple testing, and the genome-wide significance threshold at 5% was *p* < 1.11 × 10^−7^ (0.05/450062). Manhattan and QQ-plots were drawn using CMplot [40].

The genetic correlation between pairs of traits was estimated using bivariate REML implemented in GCTA v.191.4. beta3 [39]. Variance components were estimated using restricted maximum likelihood analysis (REML) implemented in GCTA v.191.4. beta3 [39], while heritability (h^2^) was calculated using REML estimates as $\frac{\sigma_{g}^{2}}{\sigma_{g}^{2}+\sigma_{e}^{2}}$ and its variance. 

The genetic contribution of SNPs was estimated using a Bayesian mixture model implemented in BayesR software [41] (https://github.com/syntheke/bayesR) that fitted all markers simultaneously with four posterior distributions for each marker. The method assumes the SNPs in the mixture model to be normally distributed and that the SNP effects are derived from a combination of four distributions with effect size proportions between 0 and 1 (i.e., explaining 0, 0.01, 0.1, and 1% of the genetic variance). For mixing, a chain length of 50,000 samples was used with the first 20,000 samples being discarded as burn-in [42]. 

### 2.4. Gene-Set Enrichment Analysis and Protein–Protein Interaction Analysis

The analysis was performed following the method described by Dadousis et al. [13,21]; briefly, for each trait a nominal *p* < 0.005 was used to filter for SNPs from the GWAS analysis. Using the SNP ID’s, gene names assigned to SNPs were filtered from the VCF file that was previously annotated with SnpEff version 4.3 [35]. The genes were then assigned to functional categories using the Gene Ontology (GO) [43] database under biological process and molecular function categories, and the Kyoto Encyclopedia of Genes and Genomes (KEGG) [44] pathway database. The gene-set enrichment analysis was performed using *goseq* R package [45]. A Fisher’s exact test was performed to test for overrepresentation of the genes in each function/pathway (gene-sets) and a false discovery rate (FDR) correction (5%) was applied to account for multiple testing. The background for the GSEA analysis was all the genes to which the SNPs used in the GWAS was annotated to (14,267 genes).

Finally, a protein–protein interaction network (PPIN) analysis was performed using the STRING database [46]. The PPIN analysis was limited to high confidence interactions with scores > 0.99; the network was clustered with an MCL with the default inflation parameter. The KEGG pathway enrichment of the PPI network was carried out using ClueGO v.2.5.5 [47]. 

## 3. Results and Discussion

Regulatory SNPs that control gene expression and genetic variants that cause protein coding changes can contribute to phenotypic variation. However, since complex traits are controlled due the additive genetic effects of a large number of genes that have small effects, several of these SNPs fail to reach the stringent thresholds adopted in GWAS to control for multiple testing. Therefore in this study, we performed a regulatory SNP GWAS (promoter and non-synonymous protein coding SNPs) and complemented it with GSEA and PPIN analysis to understand the genetic contribution and regulatory role of these SNPs on four carcass traits (BFT, CWT, EMA, and MS) in Hanwoo cattle. 

### 3.1. Phenotypes and Genomic Heritability Estimates

**Figure 1:**
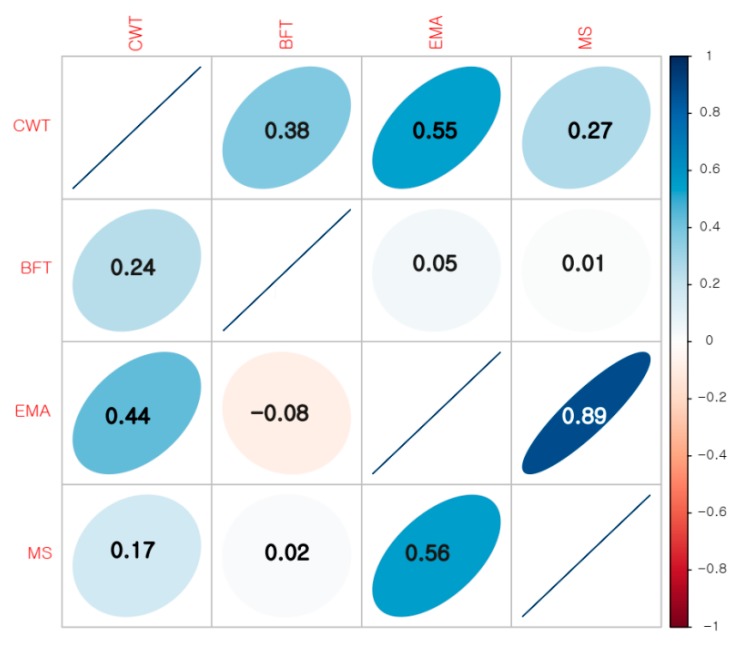
Phenotypic (upper diagonal) and genetic (lower diagonal) correlation among carcass traits in Hanwoo. The genetic correlation was calculated using bivariate reml in GCTA. The colors represent the strength of the correlation given in the scale next to the plot.

The carcass traits of the sample were normally distributed (Table 1). The mean values of CWT, BFT, EMA, and MS measured at a similar slaughter age were consistent with those of Roh et al. [48] but differed marginally with [49]. Several previous studies have reported lower estimates for these traits [29,50], which could be attributed to differences in the number of animals investigated or nutritional status during finishing period or due to differences in the methods employed for measuring the phenotypes. The lower phenotypic CV for CWT and EMA (12% and 13%) and higher estimates for BFT and MS (35% and 31%), are consistent with previous studies [29,51]. The Genetic and phenotypic correlation between traits are given in Figure 1; the phenotypic correlation among traits was stronger than their respective genetic correlation. CWT was genetically and phenotypically positively correlated with BFT, EMA, and MS, with the strongest correlation between CWT and EMA, whereas BFT had a low phenotypic correlation with EMA and MS, while it had a low negative genetic correlation with EMA. MS was highly positively correlated with EMA, both phenotypically and genetically. The high positive correlation between CWT and EMA, both genetically and phenotypically, is consistent with previous reports in Hanwoo [29,50,52,53]; similar correlations have been reported in Brahman and Japanese Brown cattle [54,55]. Kim et al. [51] had also reported a medium negative correlation between EMA and BFT. The genetic and phenotypic correlation estimated between MS and EMA was higher than what was reported previously by Roh et al. and Son et al. [48,56] but consistent with the estimates of Hwang et al. [49]. These results suggest that a selection based on CWT will have a low to medium improvement in BFT, EMA, and MS, while a selection based on BFT or MS will have minimal improvement on BFT. 

The proportions of genomic variance attributed to the regulatory SNPs were found to be 0.29, 0.25, 0.29, and 0.38 for BFT, CWT, EMA, and MS, respectively (Table 1). This is between 44% and 77% of the heritability estimated in a previous study using 11.2 million SNPs from across the genome of Hanwoo [1], suggesting that the regulatory SNPs might have a large effect on the traits evaluated in this study. This is consistent with previous reports that had reported that the missense variants (non-synonymous variants) are able to capture a large proportion of the genetic variance in beef and dairy cattle [12,57]. The genic region, i.e., the protein coding regions, were also able to capture more genetic variances than other regions for complex traits in humans [58]. 

### 3.2. Genome-Wide Association Study

**Figure 2:**
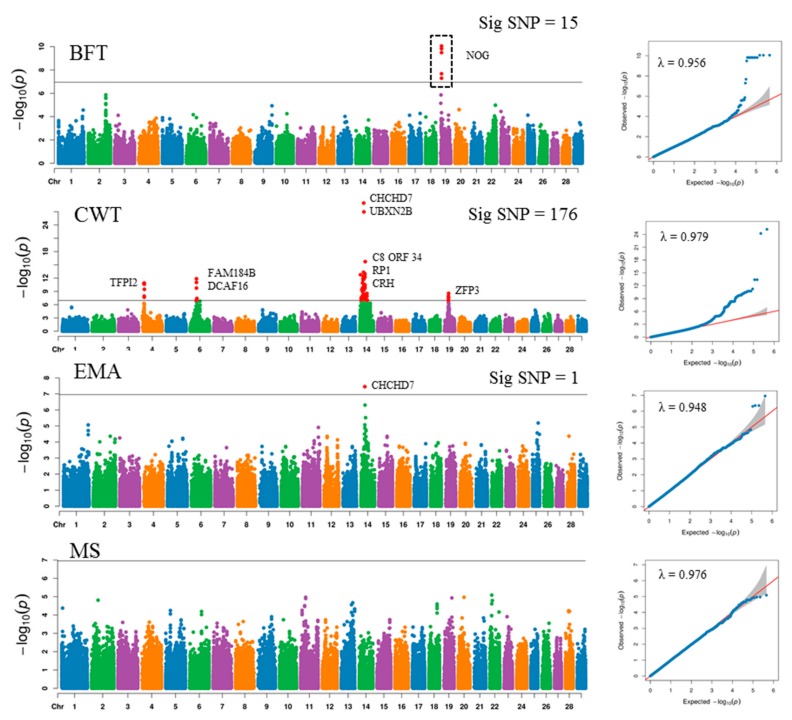
Manhattan plots of mapped single nucleotide polymorphism (SNP) markers associating with each trait, where the *Y*-axis defines the –log10 (*p*)-value against their respective positions on each chromosome (*X*-axis).

The GWAS was performed with 450,062 regulatory SNPs (protein coding, promoter SNPs, and non_synonymous SNPs) to find regions associated with the four traits studied. The mixed linear model-based GWAS revealed that 15, 176, and 1 SNPs were significantly associated with BFT, CWT, and EMA, respectively (Figure 2, Supplementary Table S1). The significantly associated SNPs for BFT ware located on BTA19, BTA4, BTA6, BTA14, and BTA19 for CWT; and for EMA on BTA 14. No significant loci were detected for MS. These significant regions were harbored by 4 (BFT), 115 (CWT), 7 (EMA), and 2 (MS) genes (Table 2). The most significant SNPs were rs109467607 (BFT), rs210030313 (CWT), and rs210030313 (EMA) located in NOG (Noggin) and CHCHD7 (Coiled-coil-helix-coiled-coil-helix Domain Containing 7) (CWT and EMA) (Figure 2, Table 2). The majority of the significantly associated SNPs for BFT were located on a 3.933 Kb region on BTA19 spanning the NOG gene for BFT; all the 15 significant SNPs on this gene were promoter SNPs.

The most significantly associated SNPs for CWT was located in a 42.01 Mb region on BTA14, which included genes such as CHCHD7, UBXN2b (UBX Domain Protein 2B), C8orf34 (Chromosome 8 Open Reading Frame 34), and TRIM55 (Tripartite Motif Containing 55). 

**Figure 3:**
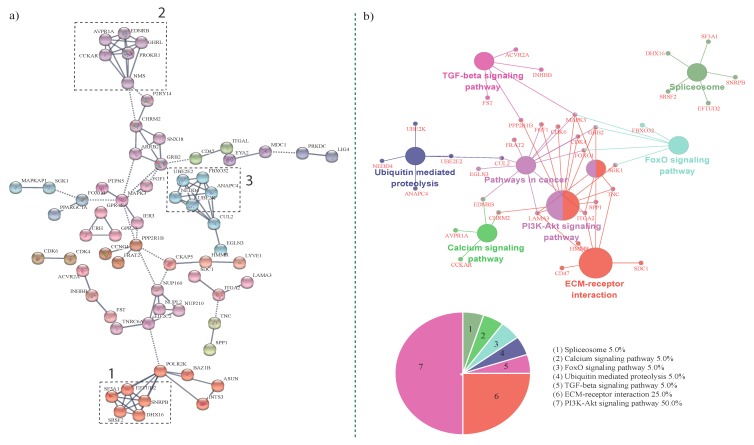
(**a**) Protein–protein interaction network analysis of genes harboring significantly associated SNPs (*p* < 0.005). Genes that were shared between traits were used for the PPIN analysis. (**b**) The KEGG pathway-based network analysis of genes within the PPI network.

A previous GWAS with 50K and 777K data have revealed major QTL for CWT on BTA 14, BTA4, and BTA6 in Hanwoo [1,8]; here we also detected significant QTLs on BTA17 and BTA19. Among the most significant SNPs for CWT on BTA14 were variants located in CHCHD7, UBXN2b, C8or34, FAM184B, TRIM55, POLR2K, CYP7A1, SDCBP, PRKDC, TOX, and PLAG1. Variants around PLAG1, CHCHD7, UBXN2B, FAM184B, and TOX have been previously reported to be associated with CWT [8,59], stature [60], live weight [61], reproductive traits [62], and puberty [63] in Hanwoo, Japanese black cattle, Nellore cattle, and Brahman cattle. A few of these loci were also associated with EMA (Figure 3). Most of these variants were all 5′ upstream promoter variants, and the large number of variants over several genes suggests a synergistic effect for the major QTL on BTA14 for CWT in Hanwoo, confirming the findings of Bhuiyan et al. [1]. Among the significantly associated SNPs for CWT, 9 SNPs were non _synonymous SNPs; these were located on TBC1D31 (TBC1 Domain Family Member 31), SPIDR (Scaffold Protein Involved in DNA Repair), PRKDC (Protein Kinase, DNA activated Catalytic Subunit), DNAJC5B (DNAJ Heat Shock Protein Family (Hsp40) member C5 β), CRH (Corticotropin Releasing Hormone), ADHFE1 (Alcohol Dehydrogenase Iron Containing 1), and NCAPG (Non-SMC Condensin I complex Subunit G). These included rs449968016 and rs41726906 on BTA14 and rs109570900 on BTA6. NCAPG, which is involved in chromatin condensation [64], has been found to be associated with CWT and body frame size [65,66]. Mutations in this gene has been implicated with cattle growth in three cattle populations [65,67]. Previous studies had not detected any significant QTL for BFT in Hanwoo [1,8]; however, we detected a significant QTL on BTA19, nearby the NOG (Noggin) gene, which induces stem cell adipogenesis [68] and was found to be associated with meat quality traits in Nellore cattle [69]. 

### 3.3. Contribution of Genomic Variants

The SNP effects was estimated using BayesR, to understand the genetic architecture and the proportion of variance explained the SNPs in each of the four distributions (with the variance 0*$\;\sigma_{A}^{2}$, 10^−4^*$\;\sigma_{A}^{2}$, 10^−3^*$\;\sigma_{A}^{2}$, and 10^−2^*$\;\sigma_{A}^{2}$). The results are given in Table 2 and Table 3. The SNPs that had the largest effects for the traits analyzed were located on BTA3 and BTA19 (BFT), BTA6 and BTA14 (CWT), BTA14 (EMA), and BTA 11 (MS) (Supplementary Figure S1). The effects were small, with ~98% of the analyzed SNPs having close to zero effects, with the rest having different degrees of genetic contribution to the traits studied. The percentage of SNPs that had the largest effect (10^−3^*$\;\sigma_{A}^{2}$ and 10^−2^*$\;\sigma_{A}^{2}$) varied between 0.001%–0.04% but they explained only 16.06%–50.83% of the total genetic variance. The effect size estimated are in agreement with previous reports [1,42,70] that reported a large proportion of SNPs contributing a zero to close to zero effect, and the infinitesimal theory. The number of SNPs having a large effect size varied between 5050 (CWT) and 8524 (MS), indicating the polygenic nature of these traits. The effect sizes of the top markers are given in Supplementary Table S2. The SNPs with the largest effect size for the traits analyzed were rs109974824 on BTA19 for BFT, rs109062047 on BTA6 for CWT, rs210030313 on BTA14 for EMA, and rs136161587 on BTA11 for MS. 

### 3.4. Gene-Set Enrichment and Protein–Protein Interaction Network Analysis Analyses

Though several regulatory SNPs were found to be significant for CWT, very few SNPs reached the significance threshold for other traits. Therefore, we decided to supplement the GWAS analysis with GSEA and PPIN analyses. Out of the 450,062 SNPs tested in GWAS, 348,088 were located in annotated genes or within 5 Kb windows upstream of the genes. In total, 14,267 background genes were annotated (Figure 1) in the *Bos taurus* UMD3.1 assembly. A total of 2657, 3064, 2261, and 2362 SNPs had a nominal *b* < 0.005 (Table 4) for BFT, CWT, EMA, and MS, respectively. They were mapped to 759, 731, 626, and 628 genes (Supplementary Table S2). GSEA showed that 25 GO and 18 KEGG terms were significantly enriched (Table 5). Genes involved in positive regulation of transcription from the RNA polymerase II promoter, Neuron projection development, Phospholipase activity, Extracellular matrix binding, and calcium ion binding ABC transporters were enriched amongst BFT associated SNPs, while regulation of cell proliferation, cell adhesion, the PI3K-Akt signaling pathway, Calcium signaling pathway, and cell cycle were enriched among genes harboring SNPs associated with CWT. Valine, leucine and isoleucine degradation, folate biosynthesis, glycerophospholipid metabolism, choline metabolism, and the insulin receptor signaling pathways were enriched amongst genes harboring SNPs associated with MS. For EMA, the associated SNP-bearing genes for cell cycle, the p53 signaling pathway, cell adhesion molecules, cell adhesion, and blood vessel development were enriched.

Since a large number of promoter SNPs were used in this study, we performed a PPIN (protein–protein interaction network) analysis using all the genes previously used for GSEA, to identify significant SNP harboring genes that physically interact (“Functional epistasis”). Sixty-five genes were found to interact (Figure 3a) through the PPIN analysis. These three clusters were found to have a high degree of interaction. Cluster 1 (Figure 3a) included SF3A1 (Splicing factor 3A subunit 1), EFTUD2 (Elongation Factor Tu GTP Binding Domain Containing 2), SNRPB (Small nuclear Ribonucloprotein Polypeptides B and B’), DHX16 (DEAH-box helicase 16), and SRSF2 (Serine and Arginine Rich Splicing Factor 2); they are all spliceosomal proteins (Figure 3b), indicating their role in post translational modification. The second cluster included members of the AVPR1A (Arginine Vasopressin Receptor 1A), EDNRB (Endothelin receptor type B), GHRL (Ghrelin and Obestatin Prepropeptide), PROKR1 (Prokineticin Receptor 1), NMS (Neuroleptic malignant syndrome), and CCKAR (Cholecystokinin A receptor); they are part of the calcium signaling pathway. In turn, the third cluster included members of the ubiquitin-mediated proteolysis pathway; they were UBE2E2 (Ubiquitin Conjugating Enzyme E2), FBXO32 (F-Box Protein 32), ANAPC4 (Anaphase Promoting Complex Subunit 4), NEDD4 (E3 ubiquitin-protein ligase), UBE2K (Ubiquitin conjugating enzyme E2K), and CUL2 (Cullin 2). Genes in this pathway have been previously found to be associated with growth and carcass traits in cattle [71]. Ubiquitin-mediated proteolysis ensures cell survival through protein turnover, and ubiquitination is also essential for signal transduction, endocytosis, and chromatin rearrangement and repair [72,73]. Functional enrichment analysis of the genes in the cluster showed that they were part of the calcium signaling, spliceosome, and ubiquitin-mediated proteolysis pathways. Genes belonging to the TGF-β signaling pathway, pathways in cancer, PI3KT signaling pathway, and ECM receptor interaction pathways were also enriched (Figure 3b).

### 3.5. Calcium Signaling Pathway

GSEA and PPIN revealed that calcium-related processes such as calcium ion binding, cellular calcium ion homeostasis, and the calcium signaling pathways were amongst the enriched terms. In total, 109 calcium-related genes were part of the gene set (Table 4, Figure 3). Calcium plays an important role in meat tenderization, feed efficiency, and muscle contraction, and several genes involved in calcium-related processes were also found to affect meat quality in Angus cattle [74,75]. Calcium signaling is also key for regulating muscle growth is beef cattle [76]. Moreover, the calpain/calpastatin system, which is a key regulator of meat tenderness and is associated with carcass and meat quality traits such are tenderness, flavor and juiciness, and marbling score, [77,78,79,80,81] is calcium dependent. Some key calcium signaling pathway genes enriched were AVPR1A (Arginine vasopressin Receptor 1A), CCKAR (Cholecystokinin A), CHRM2 (Cholinergic Receptor Muscarinic 2), EDNRB (Endothelin Receptor Type B), GHRL (Ghrelin and Obestatin Prepropeptide), PROKR1 (Prokineticin Receptor 1), and NMS (Neuromedin-S). Polymorphisms in several of these genes were found to be associated with meat quality and productivity traits [82,83,84].

### 3.6. ECM Receptor Interaction, PI3K-Akt Signaling, and Pathways in Cancer

The extra cellular matrix (ECM) is critical for tissue architecture and is involved in adipogenesis [85]. ECM comprises of a mixture of macromolecules, including glycosaminoglycans and fibrous proteins such as lammin, elastin, collagen, and fibronectin [85]. Several ECM-related terms, such as cell adhesion and extra cellular matrix interaction, were also enriched (Table 4). ECM receptor interaction has been previously implicated in adipogenesis and meat tenderness and was found to be upregulated in subcutaneous fat and intramuscular fat [86]. The PI3K-Akt signaling pathway plays a central role in controlling skeletal muscle mass and metabolism by increasing protein synthesis together with inhibition of protein degradation [87,88]. Members of the PI3K-Akt signaling pathway were enriched amongst cattle with a larger eye muscle area and also affected their intramuscular fatty acid content [89,90]. Several genes that function in cellular proliferation and cell division were part of pathways in cancer, including GHRL (Ghrelin and obestatin Prepropeptide), polymorphisms which are associated with growth and economically important traits in beef cattle [91,92].

These pathways shared several genes, and some key genes enriched included SDC1 (Syndecan 1), HMMR (Hyaluronan Mediated Motility Receptor), CD47 (Cluster of Differentiation 47), ITGA1 (Integrin α-1/β-1), LAMA3 (Laminin subunit α 3), SPP1 (Secreted phosphoprotein 1), CHRM2 (Cholinergic Receptor Muscarinic 2), TNC (Tenascin C), SGK1 (Serine/glucocorticoid-regulated kinase 1), FOXO1 (Forkhead Box O1), GRB2 (Growth factor receptor-bound protein 2), MAPK3 (Mitogen activated protein kinase 3), FGF1 (Fibroblast growth factor 1), PPP2R1B (Protein Phosphatase 2 Scaffold Subunit Abeta), EGLN2 (Egl-9 Family Hypoxia Inducible Factor 2), and EGLN3 (Egl-9 Family Hypoxia Inducible Factor 3); several of them have been found to be associated with marbling and other carcass traits in cattle [91,93,94,95,96,97,98].

### 3.7. Other Important Pathways and Terms Enriched

Several other important pathways were enriched. These included the insulin receptor signaling pathway, glycerophospohlipid metabolism, choline metabolism, and cell cycle. Insulin has a critical effect on adipogenesis [99]. The genes enriched included FOXO1, a forkhead box transcription factor, which plays an important role in energy metabolism, stress resistance, apoptosis, and cell cycle arrest; polymorphisms in this gene are associated with growth traits in Qinchuan cattle [100].

Glycerophospholipid are a major class of complex lipid with an esterified glycerol backbone, two fatty acids, and a polar head group [101]. Glycerophospholipid metabolism regulates beef fatty acid content and affects beef taste. GPD2 (glycerol-3-phophate dehydrogenase 2), which is involved in glyerophospholipid metabolism, was previously found to be associated with marbling score in Hanwoo cattle [93,102]. Choline is an essential nutrient that improves lipogenesis [103]. PLCG1 (Phospholipase C, gama 1), a gene involved in choline metabolism, was found to be significantly associated with carcass traits in Hanwoo [104].

## 4. Conclusions

The pathways and genes identified in this study enrich our standing of the molecular mechanisms underlying complex traits in Hanwoo. The candidate SNPs identified to be associated with the evaluated traits will help in breeding Hanwoo cattle with superior carcass traits. Our result shows that the regulatory SNPs are able to capture a large proportion of the total genetic variation. The genes in the associated pathways identified in this study, such as calcium signaling, ECM receptor signaling, PI3K-Akt signaling, regulation of cell proliferation, insulin signaling, glycerophospholipid, and choline metabolism, might be good candidates for identifying markers that might be associated with carcass traits in cattle. Integrating gene expression data along with the regulatory SNPs used in this study might help in identifying genes and SNPs that have a significant effect on carcass traits.

## Figures and Tables

**Table 1:** Descriptive summary of phenotype data and results of variance component estimation.

Traits	Phenotypic Data					Variance Components		
	Mean	SD	Max	Min	CV	σ^2^_g_	σ^2^_p_	h^2^
Backfat Thickness (mm)	14.08	4.9	43	2	0.35	6.79	16.98	0.29
Carcass Weight(kg)	441.22	52.7	682	159	0.12	660.25	1962.58	0.25
Eye Muscle Area (cm2)	95.8	12.0	155	34	0.13	38.23	95.69	0.29
Marbling Score (1–9)	6.1	1.9	9	1	0.31	1.24	2.04	0.38

σ^2^_g_—additive genetic variance; σ^2^_p_—phenotypic variance; h^2^—heritability.

**Table 2:** Top 5 SNPs associated with carcass traits in Hanwoo.

Trait	SNP_ID	Chr	Position	*p-Value*	SNP Effect	Gene	Type
Backfat	rs109467607	19	7617020	8.89E-11	0.0579	NOG	Promoter
Thickness	rs110172746	19	7617964	8.89-11	0.0323	NOG	Promoter
	rs109749187	19	7618889	8.89E-11	0.00256	NOG	Promoter
	rs110056766	14	7616793	1.50E-10	0.0354	NOG	Promoter
	rs109266249	19	7617322	1.50E-10	0.0311	NOG	Promoter
Carcass	rs210030313	14	25052440	4.12E-30	0.0849	CHCHD7	Promoter
Weight	rs209809798	14	26269386	1.07E-27	0.002	UBXN2B	Promoter
	rs210867053	14	34422595	2.82E-16	0.0006	C8orf34	Promoter
	rs210421179	14	34425147	2.82E-16	0.001	C8orf34	Promoter
	rs208243667	14	23986995	2.33E-14	0.004	RP1	Promoter
Eye Muscle Area	rs210030313	14	25052440	3.54E-08	0.4121	CHCHD7	Promoter
	rs209809798	14	26269386	5.00E-07	0.06	UBXN2B	Promoter
	rs41734611	14	29827599	3.05E-06	0.007	YTHDF3	Promoter
	rs108943384	14	29828860	3.05E-06	0.0097	YTHDF3	Promoter
	rs110364554	14	29829523	3.05E-06	0.0051	YTHDF3	Promoter
Marbling Score	Novel	22	11888027	8.23E-06	8.49E-03	ACVR2B	Non-synonymous
	rs109436056	20	36013931	1.08E-05	9.17E-03	EGFLAM	Non-synonymous
	rs109353762	11	30965508	1.08E-05	5.42E-03	ENSBTAG00000027015	Promoter
	rs109921982	19	50470876	1.17E-05	3.40E-03	ZNF750	Promoter
	rs109995364	11	30963318	1.27E-05	6.31E-03	ENSBTAG00000027015	Promoter

**Table 3:** Proportion of variants in each of the four distributions identified from BayesR analysis.

Trait	nSNPs	$\sigma_{g}^{2}$	Number of SNPs in Mixture Component			
			0 × $\sigma_{A}^{2}$	10^−4^ × $\sigma_{A}^{2}$	10^−3^ × $\sigma_{A}^{2}$	10^−3^ × $\sigma_{A}^{2}$
Backfat Thickness	8085	7.02	441,977 (98.2)	7920 (1.76) {5.57}	157 (0.03) {1.08}	8 (0.002) {0.37}
Carcass Weight	5050	606.40	445,011 (98.9)	4875 (1.08) {298.25}	148 (0.03) {87.07}	28 (0.01) {221.21}
Eye Muscle Area	8118	43.53	441,944 (98.20)	7927 (1.76) {34.47}	186 (0.04) {7.93}	5 (0.001) {1.04}
Marbling Score	8524	1.37	441,538 (98.11)	8361 (1.86) {1.15}	161 (0.04) {0.21}	2 (0.01) {0.01}

nSNPs—Number of SNPs; $\sigma_{g}^{2}$—total genetic variance explained by the SNPs. Values in parenthesis denotes the proportion of SNPs in that particular mixture component. $\sigma_{A}^{2}$—genetic variance. The sum of squares of the SNP effects for the particular mixture component is given in { } brackets.

**Table 4:** Number of significant SNPs identified from the genome-wide association study (GWAS) and the genes that were mapped to traits.

Traits	No. of Suggestive SNPs (*p* < 0.005)	No. of Genes Mapped within 5Kb of Suggestive SNPs
Backfat Thickness	2657	759
Carcass Weight	3064	731
Eye Muscle Area	2261	626
Marbling Score	2362	628
Background ^c^	450,062	14,267

PA—Pathway Analysis. ^c^ Background refers to the total number of SNPs used in the GWAS.

**Table 5:** Gene Ontology (GO) terms and KEGG pathways significantly enriched using genes associated with traits.

Trait	Category	Term_ID	Term	Count	%	*p-Value*	Genes	Fold Enrichment
**BFT**	BP_DIRECT	GO:0045944	Positive regulation of transcription from RNA polymerase II promoter	38	5.34	0.002	MEF2C, GDF2, NOG, FOXA2, CREM, F2RL1, PRKDC, NUFIP1, FLCN, NR2C2, KDM1A, TCF20, GALR3, HEY2, PKD2, PSIP1, POU4F2, NFATC4, TAF9, SPIC, FGF1, PIK3R1, CYR61, NFATC1, TWIST1, NPAS4, FZD5, DDX5, SREBF2, LPIN3, ACVR2A, ZMIZ2, CAMK1, BMP7, NR5A2, PBX2, ATAD2B, IL2	1.71
	BP_DIRECT	GO:0031175	Neuron projection development	10	1.41	0.002	NCAM1, GPRIN1, PTPRM, RAC3, CAMSAP2, STMN4, MAP4, LAMB1, AGER, FRY	3.62
	BP_DIRECT	GO:0007200	Phospholipase C-activating G-protein coupled receptor signaling pathway	7	0.98	0.007	C3AR1, PLCE1, LTB4R, GALR3, LTB4R2, HTR1F, F2R	4.07
	MF_DIRECT	GO:0042626	ATPase activity, coupled to transmembrane movement of substances	7	0.98	0.009	ABCA10, TAP2, TAP1, ABCB6, ABCA5, ABCA12, ABCG2	3.84
	MF_DIRECT	GO:0050840	Extracellular matrix binding	5	0.70	0.009	DMP1, OLFML2A, THBS1, CYR61, SPP1	5.87
	MF_DIRECT	GO:0005509	Calcium ion binding	34	4.78	0.014	MYL3, SYT2, EFCAB3, MYL10, SYT6, KCNIP4, MMP24, CAMKK2, SMOC2, PLCB4, CD93, EEF2K, TPT1, PKD2, HEG1, THBS1, IHH, PNLIPRP2, CRTAC1, NCALD, CDHR2, MMP16, PCDH7, PKD2L1, CDH12, THBD, CALM, EFHB, RYR3, NOTCH4, PDCD6, LRP4, CASQ2, VLDLR	1.53
	MF_DIRECT	GO:0031681	G-protein β-subunit binding	3	0.42	0.020	F2RL1, ARF6, F2R	12.91
	KEGG_PATHWAY	bta04512	ECM-receptor interaction	11	1.55	0.001	CD47, SDC1, CD36, ITGA8, COL6A2, ITGA2, LAMB1, THBS1, SV2C, HMMR, SPP1	3.56
	KEGG_PATHWAY	bta02010	ABC transporters	7	0.98	0.003	ABCA10, TAP2, TAP1, ABCB6, ABCA5, ABCA12, ABCG2	4.69
	KEGG_PATHWAY	bta03450	Non-homologous end-joining	4	0.56	0.010	DCLRE1C, PRKDC, NHEJ1, MRE11	8.66
	KEGG_PATHWAY	bta04080	Neuroactive ligand-receptor interaction	18	2.53	0.030	GABRG1, C3AR1, F2RL1, VIPR2, CRHR1, EDNRB, CHRM2, LTB4R, GALR3, P2RY14, AVPR1B, LTB4R2, CNR2, HTR1F, GLP1R, GHR, OPRD1, F2R	1.73
**CWT**	BP_DIRECT	GO:0042127	Regulation of cell proliferation	11	1.58	0.038	SGK1, TNFRSF11B, SGK3, TNC, JTB, EGLN3, PKD2, GHRL, TNK1, NDRG1, RPA3	2.09
	BP_DIRECT	GO:0007155	Cell adhesion	12	1.72	0.047	IBSP, NOV, PARVG, ITGAL, CD47, OPCML, TNC, SULF1, GP1BA, GRHL2, CTNNA3, SPP1	1.92
	MF_DIRECT	GO:0044822	Poly(A) RNA binding	45	6.46	0.010	YWHAZ, ASS1, GRB2, NOC3L, PRKDC, KNOP1, RPS19BP1, NUFIP2, RPS27, PRR3, RPL7, MAK16, MACF1, DHX37, SND1, NUDT16L1, PSIP1, DHX16, RBM47, RPL12, RPS20, CDC42EP4, TNRC6A, MTERF1, KHDRBS3, MRPS28, ZC3H15, TBL2, TSR1, MAGOH, EFTUD2, PKN2, RPL26, CASC3, NUPL2, RBBP6, FLNB, CMSS1, SRSF2, SYNE1, PTCD3, POP1, DDX31, DNTTIP2, KCTD12	1.46
	MF_DIRECT	GO:0005096	GTPase activator activity	14	2.01	0.017	PREX2, ASAP2, ARHGAP24, RGS22, ARHGAP31, RGS20, RABEP1, ARHGAP42, TBC1D1, CDC42EP4, RAP1GAP2, ARAP2, CDC42EP3, TBC1D20	2.09
	MF_DIRECT	GO:0005509	Calcium ion binding	31	4.45	0.029	NKD1, CLSTN3, EFCAB5, DUOX2, MMP27, MMRN1, ZZEF1, KCNIP4, SMOC2, MACF1, FAT3, CRB2, PLA2G12A, EFCAB1, PKD2, SRR, PLCB2, HPGDS, NCALD, PCDH8, DLL1, PCDH7, SLIT2, PCDH18, ATP2C1, RYR3, NUCB2, SULF1, SCIN, ANXA13, ADGRL4	1.48
	KEGG_PATHWAY	bta04151	PI3K-Akt signaling pathway	21	3.01	0.021	PPP2R1B, FGF6, IBSP, CRTC2, YWHAZ, SGK1, SGK3, GRB2, TNC, PKN2, CDK6, GNG11, NFKB1, GNGT1, LAMA3, MAPK3, PDGFRA, PIK3R5, FGF1, MYC, SPP1	1.70
	KEGG_PATHWAY	bta04713	Circadian entrainment	9	1.29	0.021	GNGT1, ADCY8, GRIA1, RYR3, MAPK3, CACNA1I, GNG11, CACNA1C, PLCB2	2.61
	KEGG_PATHWAY	bta04020	Calcium signaling pathway	12	1.72	0.071	CCKAR, ADRB1, P2RX1, ADCY8, PHKG1, RYR3, CACNA1I, PDGFRA, AVPR1A, PPP3CA, CACNA1C, PLCB2	1.78
	KEGG_PATHWAY	bta04110	Cell cycle	9	1.29	0.075	YWHAZ, RAD21, ANAPC4, BUB1, PRKDC, CDK6, MYC, BUB3, STAG1	2.02
**MAR**	BP_DIRECT	GO:0016477	Cell migration	11	1.82	0.006	CUL3, TNS3, ERG, SDC1, FSCN2, PLCG1, IL12A, SIX2, IL12B, BAMBI, SRMS	2.79
	BP_DIRECT	GO:0042127	Regulation of cell proliferation	11	1.82	0.024	TNFRSF6B, ITK, SGK2, BIRC7, EGLN3, GHRL, TOPORS, TFAP2C, LGR5, SRMS, NKX2-3	2.25
	BP_DIRECT	GO:0008286	Insulin receptor signaling pathway	5	0.83	0.037	SLC2A8, PDK2, FOXO1, RHOQ, ZNF106	3.94
	BP_DIRECT	GO:0006874	Cellular calcium ion homeostasis	6	0.99	0.058	EDN3, ATP2C2, ATP2C1, PKHD1, CCL8, ATP13A3	2.85
	MF_DIRECT	GO:0005524	ATP binding	50	8.28	0.086	MLH1, SKIV2L2, ACSF2, MTHFD1L, LONP1, PIP5KL1, DDX28, PRKACB, SGK2, MYH3, OLA1, CFTR, LIG4, CDK4, NEK11, CDKL4, MAST3, ATP2C2, ACVR2B, DHX29, ATP2C1, NEK7, XYLB, DNAH9, SPO11, KIT, ITM2B, STK40, VRK3, MAP3K1, ENTPD8, LMTK3, ITK, PDK2, SMCHD1, AK1, TGFBR2, TRIO, ATP1A1, STRADB, ATP13A3, TRANK1, SMC4, TP53RK, TEX14, PLK2, PSMC3, MYO16, FPGS, SRMS	1.23
	MF_DIRECT	GO:0005509	Calcium ion binding	26	4.30	0.089	LALBA, GPD2, LPO, MYL2, EFCAB5, CRTAC1, PAMR1, PCDH10, CDHR3, SYT9, SYT6, PCDH8, STAB2, LPCAT2, SLIT1, ZZEF1, HMCN2, CDH12, ANXA6, EGFLAM, CLGN, FAT3, PLCG1, ATP2C1, FAT1, MCFD2	1.38
	KEGG_PATHWAY	bta04060	Cytokine-cytokine receptor interaction	17	2.81	0.000	TNFRSF6B, TGFBR2, CCL8, IL10, IL12RB2, CCR9, ACVR2B, IL17B, IL12RB1, PRLR, CCR4, IL12A, TNFRSF19, CSF3R, IL12B, IFNGR2	2.83
	KEGG_PATHWAY	bta05200	Pathways in cancer	22	3.64	0.003	RALBP1, TGFBR2, BIRC7, EGLN3, MLH1, FOXO1, LEF1, ITGA3, NFKB2, KIT, CDK4, MMP2, AGTR1, EDNRB, CUL2, PLCG1, SOS1, RALB, CSF3R, HHIP, PRKACB, TRAF6	2.00
	KEGG_PATHWAY	bta00280	Valine, leucine and isoleucine degradation	5	0.83	0.047	HMGCS2, OXCT1, DLD, IL4I1, ACAD8	3.62
	KEGG_PATHWAY	bta00790	Folate biosynthesis	3	0.50	0.048	GGH, FPGS, GCH1	8.37
	KEGG_PATHWAY	bta00564	Glycerophospholipid metabolism	7	1.16	0.050	GPD2, CHKA, ADPRM, ETNK1, LPCAT2, PLPP1, PLPP2	2.61
	KEGG_PATHWAY	bta05231	Choline metabolism	7	1.16	0.054	CHKA, PLCG1, SOS1, SLC22A5, PLPP1, PLPP2, SLC22A1	2.56
	KEGG_PATHWAY	bta04630	Jak-STAT signaling pathway	9	1.49	0.055	IL12RB2, IL12RB1, PRLR, SOS1, IL12A, CSF3R, IL12B, IFNGR2, IL10	2.16
**EMA**	BP_DIRECT	GO:0031663	Lipopolysaccharide-mediated signaling pathway	4	0.34	0.057	MAPK3, NFKBIA, PRKCE, PTAFR	4.52
	BP_DIRECT	GO:0001568	Blood vessel development	4	0.34	0.057	MEF2C, PSEN1, ITGAV, RAPGEF2	4.52
	BP_DIRECT	GO:0042127	Regulation of cell proliferation	9	0.77	0.090	SGK1, PTGS2, TNC, LCK, CHST11, NFKBIA, FAS, PLAU, TEC	1.95
	BP_DIRECT	GO:0007155	Cell adhesion	10	0.85	0.096	ITGAL, LYVE1, TNC, NPHS1, ACAN, ITGA2, GP1BA, PRKCE, FN1, MYH10	1.83
	MF_DIRECT	GO:0008168	Methyltransferase activity	6	0.51	0.004	ZCCHC4, METTL21B, TRMT10A, PRMT9, NSUN3, METTL18	5.52
	MF_DIRECT	GO:0005524	ATP binding	54	4.61	0.013	KIF22, SEPHS2, INO80, IARS2, PIP5KL1, MOS, PRKACB, SIK2, ABCE1, SGK1, MYH3, CDK6, LIG4, PRKCE, UBE2C, CDK4, CDKL4, UBE2N, MAST4, UBE2K, ATP2C1, RIPK1, LCK, MAPK3, RRM1, DNAH9, PEAK1, PRKDC, CHEK2, ITM2B, DNAH5, STK40, ENTPD8, STK38L, ABCA13, AATK, TEC, DHX8, PDK2, ALPK3, AK1, AK7, RIMKLB, TP53RK, GLYCTK, DYRK1A, ATP2A1, DGKZ, ABCC2, NLRP13, FPGS, KATNAL2, MYH10, ATAD2B	1.38
	MF_DIRECT	GO:0005509	Calcium ion binding	29	2.47	0.016	GALNT3, TBC1D9, MMP8, MMP27, C2CD4D, EDIL3, KCNIP1, CRB2, ACAN, CDH26, HPGDS, GPD2, NOX5, ADGRE3, HSPG2, S100A10, STIM1, TC2N, CABYR, CLGN, CDH17, ATP2C1, ATP2A1, DSC3, RYR2, DSC2, ANXA13, SGCA, LCP1	1.59
	KEGG_PATHWAY	bta04110	Cell cycle	11	0.94	0.005	E2F2, YWHAH, HDAC1, RBL1, ANAPC4, PRKDC, CDK6, ANAPC10, ORC6, CHEK2, CDK4	2.89
	KEGG_PATHWAY	bta04115	p53 signaling pathway	7	0.60	0.020	RFWD2, CASP3, CDK6, CHEK2, FAS, CDK4, CCNG1	3.24
	KEGG_PATHWAY	bta04514	Cell adhesion molecules (CAMs)	10	0.85	0.044	GLG1, CLDN8, ITGAL, CLDN18, CD86, VTCN1, ITGAV, CD274, NEO1, CLDN25	2.13

## Supplementary Materials

The following are available online at https://www.mdpi.com/2073-4425/11/3/316/s1, Figure S1: Manhattan plot with estimated genetic variation explained by individual SNPs for BFT, CWT, EMA and MS. Combination of normally distributed variance ranging between 0 to 1% were used for estimating SNP effects using BayesR. Table S1: List of significant SNPs (*p* < 1 × 10^−5^) associated with BFT, CWT, EMA and MS, Table S2: List of all SNPs (*p* < 0.005) associated with BFT, CWT, EMA and MS that were used for GSEA and pathway analysis.
