# Supplementary figures and images for: A Gene-Set Enrichment and Protein–Protein Interaction Network-Based GWAS with Regulatory SNPs Identifies Candidate Genes and Pathways Associated with Carcass Traits in Hanwoo Cattle
Source: Genes (Basel). 2020 Mar 16;11(3):316. doi: 10.3390/genes11030316 (PMC7140899; doi:10.3390/genes11030316)

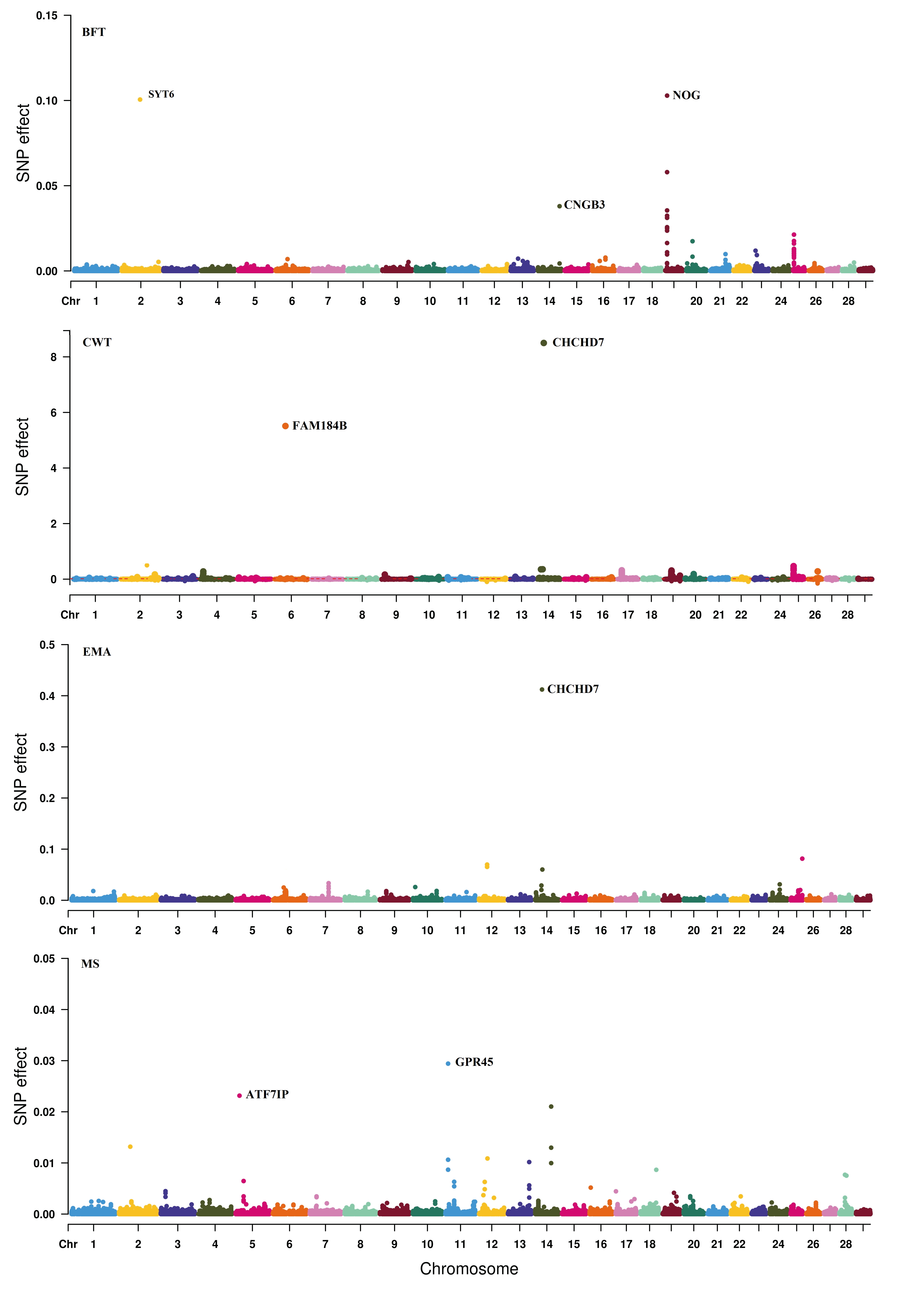

Supplement: Supplementary file 1 [file genes-11-00316-s001.zip › genes-730814-supplementary/Supplementary Figure S1.png]
